# Quality of written feedback given to medical students after introduction of real-time audio monitoring of clinical encounters

**DOI:** 10.1186/s12909-020-02158-6

**Published:** 2020-07-25

**Authors:** Michael Sanatani, Kylea Potvin, Henry Conter, Kimberly Trudgeon, Andrew Warner

**Affiliations:** 1 Department of Oncology, Division of Medical Oncology, Schulich School of Medicine & Dentistry, Western University, London, ON Canada; 2 London Health Sciences Centre, London Regional Cancer Program, PO Box 5010, 800 Commissioners Rd East, London, N6A 5W9 ON Canada; 3 Schulich School of Medicine & Dentistry, Undergraduate Medical Education, London, Canada

**Keywords:** Feedback, Direct observation, Communication skills, Workplace assessment

## Abstract

**Background:**

Direct observation is necessary for specific and actionable feedback, however clinicians often struggle to integrate observation into their practice. Remotely audio-monitoring trainees for periods of time may improve the quality of written feedback given to them and may be a minimally disruptive task for a consultant to perform in a busy clinic.

**Methods:**

Volunteer faculty used a wireless audio receiver during the second half of students’ oncology rotations to listen to encounters during clinic in real time. They then gave written feedback as per usual practice, as did faculty who did not use the listening-in intervention. Feedback was de-identified and rated, using a rubric, as strong/medium/weak according to consensus of 2/3 rating investigators.

**Results:**

Monitoring faculty indicated that audio monitoring made the feedback process easier and increased confidence in 95% of encounters. Most students (19/21 respondents) felt monitoring contributed positively to their learning and included more useful comments.

101 written evaluations were completed by 7 monitoring and 19 non-monitoring faculty. 22/23 (96%) of feedback after monitoring was rated as high quality, compared to 16/37 (43%) (*p* < 0.001) for monitoring faculty before using the equipment (and 20/78 (26%) without monitoring for all consultants (*p* < 0.001)). Qualitative analysis of student and faculty comments yielded prevalent themes of highly specific and actionable feedback given with greater frequency and more confidence on the part of the faculty if audio monitoring was used.

**Conclusions:**

Using live audio monitoring improved the quality of written feedback given to trainees, as judged by the trainees themselves and also using an exploratory grading rubric. The method was well received by both faculty and trainees. Although there are limitations compared to in-the-room observation (body language), the benefits of easy integration into clinical practice and a more natural patient encounter without the observer physically present lead the authors to now use this method routinely while teaching oncology students.

## Background

Direct observation in medical education is a necessary prerequisite activity for the provision of clinical formative feedback [1, 2], and it is thought that the main contribution to enhanced learning from direct observation occurs based on its facilitation of constructive and valid feedback, or “coaching” The combination of initially observing a trainee perform a clinical task, and then having a faculty-trainee dialogue about the current performance, comparing it to an ideally attainable goal and exploring ways to move towards that level, can be called coaching and is a commonly used conceptual framework of clinical and bedside teaching [3]. It is an integral part of competency based medical education (CBME) and introducing it to clinical teaching is one of the main challenges in the transition to CBME.

Feedback in the context of clinical coaching has thus been defined as “specific information about the comparison between a trainee’s observed performance and a standard, given with the intent to improve the trainee’s performance.” [4] Several authors have attempted to establish characteristics of feedback that would make it effective in facilitating learning, with several common themes emerging [5–7]. A systematic review of feedback confirmed that it is effective in promoting physician development, but highlighted that it is not only the content but also aspects of delivery, coach credibility, and receptivity of the trainee that inform effectiveness [8]. Nevertheless, certain content characteristics of clinical feedback, whether delivered written or verbal, appear to be universally reported as promoting effectiveness of coaching. Task-oriented feedback identifying very specific and actionable areas of improvement, relevant to agreed-upon learning goals, is felt to be stronger than feedback containing general or value statements focused on the individual’s characteristics [7]. Feedback based on actual observation of a trainee performing in a workplace environment is one of the most valued and powerful influences on a trainee [9]. Finally, feedback should be delivered in a timely fashion [7]. The role of the quality of feedback has been demonstrated in several studies. Boehler et al. demonstrated that knot-tying skills improved more with effective feedback than with praise [10], and Engerer and colleagues showed that communication skills improved more with specific feedback than with general comments [11]. Trainees also value feedback of high quality, yet surveys consistently demonstrate a low rate of feedback felt to be of good quality according to the framework outlined above.

Translating the evidence for high quality feedback into daily clinical and teaching practice means overcoming several challenges. Kogan et al. recently summarized the evidence guiding direct observation for the purposes of promoting clinical skills in trainees [12]. The observation should be of an authentic clinical encounter. Direct observation, as Kogan et al. outline, allows assessment at the “does” level of Miller’s pyramid [12]. The presence of an observer in the room, however, may give trainees the impression that assessment is in progress, even if the intended purpose is formative [13]. Others have tried to diminish this observer effect. Sehgal et al. [14] used a one-way mirror, and trainees indeed indicated they felt their patient interaction was then not adversely affected by the evaluative process. Also, feedback should not be limited to quantitative ratings [12]. This is especially important for written feedback, which is presented to competency committees and informs decisions about trainee progression through training.

In our study, we wished to explore an observation method that would not impose much additional cognitive or time load and thus be feasible for faculty to use in a busy clinic, and might still improve the quality of feedback given to trainees. We introduced remote wireless audio monitoring of third year medical students by using portable microphones and receivers (audio-only monitors) which can be readily introduced to outpatient clinics without any technical infrastructure changes. We had three objectives.

First, *what is the effect of live audio observation on the quality of written feedback given to the directly observed trainees?*

Second, *is audio monitoring an observation method which would be a feasible way to introduce limited live “observation snapshots” into a busy clinic, as determined by faculty?*

Finally, *how do trainees perceive the effects of audio monitoring on their clinical learning experience and the teaching provided to them by faculty?* In the pursuit of providing faculty the opportunity of observing a “genuine” encounter, we wish to ascertain whether audio monitoring would be perceived as intrusive. In addition, do the observed students find the faculty responses to be helpful to their learning?

## Methods

### Design and study context

Many students on the oncology rotation at our centre in the past indicated they did not find the currently provided written feedback comments helpful. Direct observation is difficult in a busy clinic, and we sought to develop an efficient yet effective method for direct observation. To ascertain whether live audio monitoring of oncology student-patient encounters was perceived as feasible by faculty, associated with high quality written feedback, and seen as valuable by students, we introduced live audio monitoring into the clinical oncology rotations for third-year medical students at Western University. As it is not clear what observation duration is “sufficient” for provision of high quality feedback, we left this at the discretion of the consultant in the interest of encouraging actual implementation in a busy clinic. While some may choose to listen to the entire encounter, even those just listening for several minutes may observe enough to move beyond the first impression judgment which often informs evaluation [15]. Faculty and trainees were surveyed afterwards, and the quality of the written feedback was independently assessed according to a rubric. The study design was non-randomized and observational, with several faculty taking part in audio monitoring and the others serving as the control.

### Participants and recruitment

Eligible trainees were in their third year of medical training at the Schulich School of Medicine & Dentistry at Western University, and enrolled in a two-week clinical oncology selective block. Typically students rotate through outpatient oncology clinics of 3–5 medical and radiation oncology consultants during this time. Information about the study was included in the routine orientation on the first day of the rotation by the rotation supervisors, and participation was voluntary without any academic consequences if students declined. If they consented to take part, students were told that any patient visit during the second week of their rotation might be live monitored via audio feed from the patient room if they worked in a monitoring consultant’s clinics, and they were aware of which consultants were monitoring consultants. Consultant assignment to the monitoring arm of the study was by volunteering rather than by randomization of all faculty, as there were at the outset insufficient faculty willing to take part in the study and potentially be randomized to the intervention arm.

### Intervention

Monitoring was done via a Williams Sound PPA T46 FM transmitter, with the battery-powered microphone and sending unit hidden in a tissue box in the patient clinic room, and the monitoring consultant clipping the receiver to their clothing or lab coat and plugging in headphones when they wanted to listen in from the workstation or hallways outside the room. Patients were made aware of the microphone in the tissue box by the nurse accompanying them into the clinic room and instructed on how to turn it off if desired. Students were not made aware that a specific room contained the microphone that day. Monitoring consultants, during the monitored week with the student, were free to use the device as they chose – either to listen to a full encounter or part of it, while physically present at their workstation outside the patient room. In our centre there are often several occasions in a busy clinic when a consultant has a few minutes of inaction waiting for a blood draw, or for a room to become available. Time spent listening to students via the audio monitor thus could be taken from previously unused minutes waiting for a clinic room.

### Data collection

Written consent was obtained from all consultants taking part in audio monitoring (“monitoring consultants”) as well as from participating third year medical students at the beginning of their oncology clinical rotation. Verbal consent was obtained from any patient being seen in a room in which the microphone was present, by the clinic nurse putting the patient in the room.

#### Written feedback quality

Written rotation feedback was completed online as per usual practice, with the addition of an initial first feedback documentation after 1 week of working with a monitoring consultant, before monitoring started during the second week. This was done to allow for longitudinal sequential comparison of feedback given by the same consultant to one student without and with the benefit of using the audio monitor.

Trainees were told which written feedback comments were based on a monitored clinic experience at their end-of-rotation evaluation meeting, and asked to comment on the perceived usefulness of the feedback they received after monitored encounters, compared to that received as per usual practice.

#### Faculty and trainee surveys

Faculty surveys were to be carried out online for each observed student. The choices were “Audio monitoring was easier or gave me more confidence that my feedback to the trainee was meaningful and actionable”, “Audio monitoring was not significantly different”, or “Audio monitoring was detrimental”. In addition, there was space to comment on the feasibility of this method of observation in a busy clinic. Observed students also completed an online survey, and the survey choices were tabulated. The choices were “Audio monitoring was more helpful”, “Audio monitoring was not significantly different”, or “Audio monitoring was detrimental”. In addition, there was space for free text comments.

### Data analysis

#### Feedback quality

Written feedback comments were de-identified and rated by two investigators (MS and HC), one of whom was not clinically involved, as strong, weak, or neither strong nor weak based on an exploratory rubric modified from Nesbitt et al. with permission [16], considering also Lefroy et al’s definitions of “DO’s” and “DON’Ts” of feedback (Table 1). A third blinded investigator who was not a monitoring consultant (KP) resolved any evaluations that were discordant between rating investigators (MS and HC). Descriptive statistics of the feedback ratings were generated for all medical students (*n* = 20), all monitoring consultants (*n* = 7) and for all evaluations (*n* = 101). Results were stratified by [1] audio monitored vs. not audio monitored and [2] encounter type (audio monitored; not audio monitored but with a monitoring consultant; not audio monitored and a non-monitoring consultant), and compared using the Chi-square test, or Fisher’s Exact test as appropriate. All statistical analysis was performed using SAS version 9.4 software (SAS Institute, Cary NC, USA) using two-sided statistical testing at the 0.05 significance level.

**Table 1:** Exploratory rating framework for written feedback – Feedback Quality Evaluation Form provided to blinded raters

**▢ Weak**	■ Lacking performance content altogether o E.g.*“Saw many cases of lung cancer”* ■ Blank ■ Nonspecific o E.g. “*A pleasure to work with”, “Functions at PGY-1 level”* ■ Irrelevant o E.g.*“Spends much time studying after clinic”* ■ Based on second-hand information o E.g. *“Caused pt in Dr. X’s clinic to cry”* ■ Predominantly evaluative without specific aim of performance improvement o E.g.*“above average level of knowledge”*
**▢ Neither weak nor strong**	■ Mentions points of good performance in general o E.g.*“Good communicator”* ■ Mentions areas for improvement in general o E.g. *“Should take more time with patients”*
**▢ Strong**	■ **Specific areas** for improvement o E.g.*“Explore symptoms in more depth during review of systems”* ■ Based on **direct observation** o E.g.*“Did not respond to patient comments about anxieties/worries on several occasions – work on questioning further to validate and explore patient concerns”* ■ Relevant to **course goals** o *“When presenting case history, lung cases were better presented than prostate cases. Review prognostic features of CA prostate important in initial consultation discussion”* ■ **Explains the gap** [between observed performance and explicit standard] o *“At this point in training would be expected to develop a differential diagnosis of at least 3 conditions or etiologies underlying a presenting symptom. Tends to focus only on the most likely cause – encouraged to think of other potential causes as well”*

#### Faculty acceptance and feasibility; student acceptance

The proportion of faculty answering each survey option was calculated. Qualitative thematic analysis was carried out on the narrative comments by faculty and by trainees according to the method of Braun and Clarke [17]. Three investigators (MS, KP, KT) independently coded themes from the comments and final coding was arrived at by consensus and tabulated.

### Ethics

Approval for the study was obtained from the Human Subjects Research Ethics Board at Western University.

## Results

Between September 2016 and April 2018, 19 non-monitoring consultants and 7 monitoring consultants completed 101 written evaluations (23 monitored, 78 not monitored) for a total of 20 medical students completing their oncology selective at the London Regional Cancer Program.

### Feedback quality

**Fig. 1:**
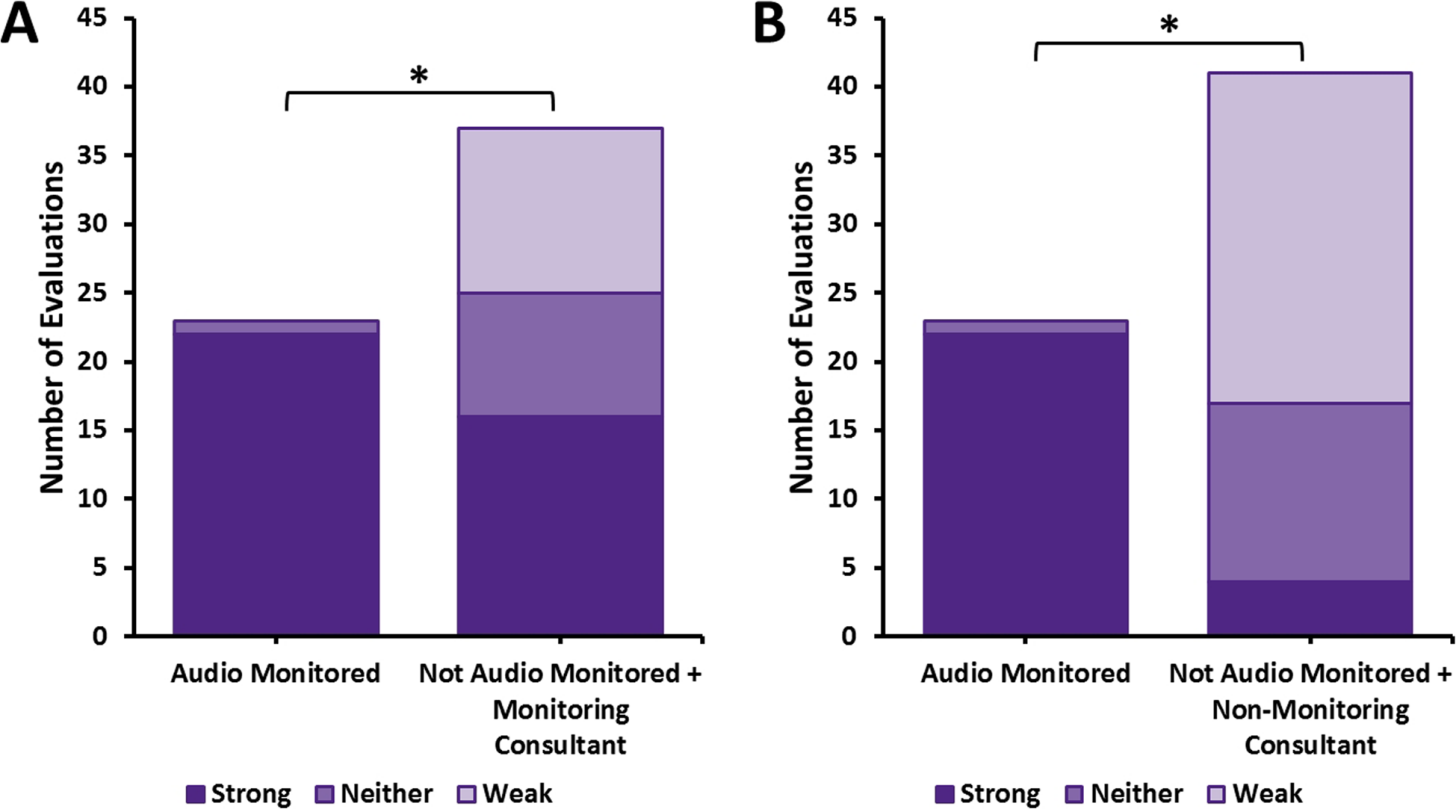
Comparison of written feedback quality after audio-monitored and non-monitored outpatient oncology rotation weeks for (**a**) monitoring consultants using (*n* = 23 ratings) versus not using (*n* = 37 ratings) audio equipment and (**b**) non-monitoring consultants (*n* = 41 ratings) versus monitoring consultants using audio equipment. Asterisk (*) indicates significant difference (*p* < 0.001) (File attached)

For evaluations with included audio monitoring of the trainee, 22/23 (96%) evaluations were rated as high quality, as compared to 20/78 (26%) for all evaluations completed without the consultant having listened in via the audio monitor (*p* < 0.001) (Fig. 1). Considering just consultants who had volunteered to be monitoring consultants, this group gave high quality feedback in 16/37 (43%) of unmonitored evaluations compared to monitored evaluations (*p* < 0.001). The blinded rating investigators agreed in 70/101 (weighted Kappa 0.69) of evaluations.

Feedback comments in unmonitored encounters were generally nonspecific, generally evaluative and with only limited explanation of what trainees could do to improve their skills. In contrast, the vast majority of feedback based on using audio monitoring was much more constructive and specific as is reflected in the ratings assigned based on our rubric. Representative comments are shown below:

*Performed well for level of training. She had an organized approach to performing a clinical assessment and asked appropriate follow-up questions based on patient answers. Her interactions with patients were empathetic and friendly. Synthesis of information could be improved by keeping in mind a differential with no more than 3 considerations in order to focus follow-up questions*. (feedback rated as “strong” - monitored)

*Average performance. Some gaps in knowledge base consistent with level.* (rated “weak”)

*Good approach. Detailed review of clinical applicable items. Thorough. Continue to build up on the present base of knowledge.* (rated as “neither weak nor strong”)

### Faculty acceptance and feasibility

Initially, there was some faculty reluctance to consider taking part in the study. However, those consultants who did become monitoring consultants overwhelmingly found the audio monitoring helpful. Amongst the monitoring consultants, out of 21 monitored evaluations for which a post-evaluation survey was completed, 20 (95%) indicated that audio monitoring made the feedback process easier or increased confidence, compared to one (5%) who indicated the audio monitoring of that encounter did not significantly increase confidence in evaluation of the student.

**Fig. 2:**
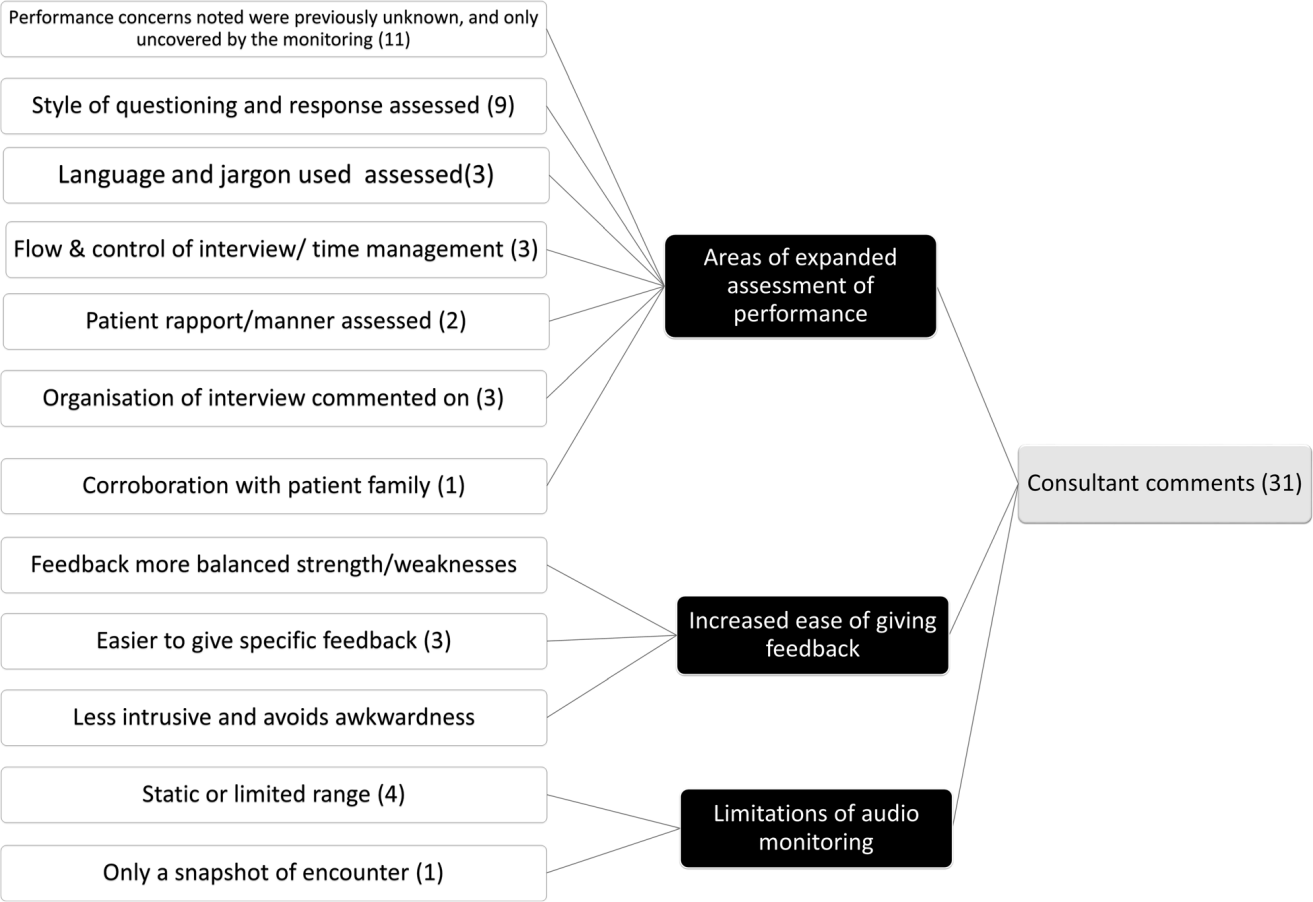
Thematic analysis of faculty comments on the use of live audio monitoring. The number of themes is larger than the number of comments analysed as some comments addressed multiple themes

Thematic analysis of the comments by monitoring faculty is shown in Fig. 2. The dominant theme was the realization that the audio monitoring allowed observation of – and thereby facilitated feedback on—areas of performance which would otherwise have been hidden.

No monitoring consultants felt that the audio monitoring was detrimental to their teaching activity or clinic workload. On the contrary, consultants appreciated observing behaviour they could give feedback on:

I specifically addressed details in his patient interview which I never would have known about without listening in. (Consultant 2)

Listening to the interaction allowed one to evaluate multiple things one could not do without the audio monitoring … Tone of voice - appropriate or not … Organization of questions and responses in patient interaction … Responses of trainee to patients answers and comments - were they responding appropriately and directly, would they say 'I'm not sure' if they did not know the answer, used language and tone conveying empathy/compassion/understanding … (Consultant 4)

### Student reception

From the students’ perspective, out of 21 encounters for which post-encounter surveys were available, the audio monitoring was reported as contributing positively to their learning experience in 19 (95%) of the monitored encounters, not adding anything significant in one (5%), and detrimental in one (5%) case (due to the student reporting feeling more nervous knowing he was being potentially observed).

**Fig. 3:**
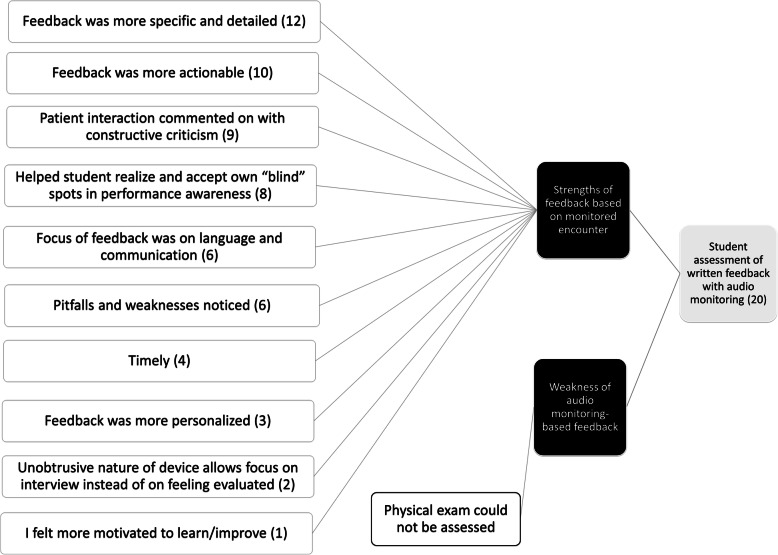
Thematic analysis of student comments on the use of live audio monitoring. The number of themes is larger than the number of comments analysed as some comments addressed multiple themes

Thematic analysis uncovered that the majority of students found the specificity of the comments from monitored encounters remarkably different from what they were used to (Fig. 3). While only three faculty members felt the specificity of their comments was increased, 12 students noted an increased specificity and 10 found the feedback more actionable. Students attributed this directly to the fact that the consultant had credible first-hand knowledge of what happened in the room:

I found that when I wasn't monitored feedback was very general, such as case presentations were well organized or read around cases/medications. When monitored, the feedback was much more specific, such as 'I enjoyed that you explained what you were doing during your physical exam' or you used leading questions like 'you aren't having any pain?' instead of 'are you having any pain?'. The physician was able to hear exactly how I was asking questions and interacting with the patients which allowed for more specific and useful feedback of how to improve my patient encounters. (Student 19)

Feedback from live monitoring was far more detailed and provided specific areas for improvement with appropriate and useful examples. Pragmatically this is very helpful, but the feedback also feels less generic … , I feel more invested in improving these areas than with the feedback without live-monitoring. (Student 8)

Another theme to emerge from the comments was the realization by the students that feedback was being given on some performance deficiencies they themselves had not known existed:

“ … they were also able to comment on some things I wasn’t even aware of doing” (Student 9)

## Discussion

In our study we demonstrated that most of the written feedback provided to students on their oncology rotations in the absence of direct observation by the majority of faculty is likely of low educational value. However, feedback given after faculty used the audio monitoring system was of significantly higher quality – more specific and more actionable, as reflected in the higher feedback quality rubric ratings. Based on the premise that higher quality written feedback promotes learning, an intervention which is associated with more specific, timely, and actionable feedback would be a valuable contribution to the improvement of clinical oncology education [7].

Watling et al. have highlighted the importance of credibility judgments made by trainees in regards to faculty feedback, and we hypothesized that students would appreciate and respect feedback they know is actually based on direct observation [18]. Student comments indeed reflect the qualitative difference in the written feedback they received after audio monitoring compared to what they were experienced previously or with non-monitoring consultants, with over half the students mentioning feedback *specificity* as a noticeable improvement. Specificity in clinical feedback is arguably only possible if behaviour has been directly observed, and the students’ appreciation of specificity in feedback comments based on audio monitoring speaks to the importance of introducing observation into clinic rotations. Students also noted how the areas of feedback often involved performance gaps they did not know existed. As Ramani et al. have elegantly outlined [19], the Johari window framework of self-awareness can be applied to feedback. Audio monitoring introduced students to behaviours which were unknown to themselves but now known to others (“accepting the blind”). Conversely, faculty discovered behaviours previously unknown (“uncovering the unknown”), a classic application of the Johari window construct to education. These themes were prominent in qualitative analysis of both faculty and student comments (Figs. 2 and 3).

To date, studies using recording of trainee-patient interactions have primarily focused on video recording of interactions and providing the recording to the trainee after the encounter for discussion [20, 21]. Direct observation with the consultant in the room has been studied in this regard and found to be of benefit in improving comfort with patient care skills, presumably due to the improved feedback and teaching given [22]. However, it does require additional time commitment from the consultant and possibly does not represent the way the trainee usually interacts with patients. Our intervention is unique in that it can be introduced without any changes to clinic infrastructure or schedules and can be used on an ad-hoc basis even if trainee assignment to a clinic changes. This flexibility is a great advantage over traditional ways of observing requiring a one-way mirror, in-room observation, or a video or hardwired audio feed installed in the patient rooms.

With the advent of competency-based education, we expect such observation infrastructure to be gradually introduced in most teaching centres over the next years to decades. However, until such time as that infrastructure is standard equipment, using a portable audio monitor may help provide trainees with significantly improved feedback particularly on their communication skills. We plan to use the audio monitors next in the postgraduate context with oncology residents to observe their systemic therapy consent conversations and see feedback given translates into better patient education outcomes.

Another future question arising from our study is whether introduction of audio monitoring for *all* faculty will result in the feedback quality improving across the department. The faculty volunteers using the monitors may have been, by virtue of their interest in medical education, already higher skilled at using audio observations to give specific and actionable feedback. It remains to be seen whether a similar effect on feedback quality would result if all faculty were to be trained on giving feedback and required to use the monitors. Finally, as Pelgrim et al. [23] point out, many studies of assessment tools and procedures examine learner attitudes towards the assessment procedure rather than their effect on learning outcomes, and our study is no exception. The question of the impact of audio monitoring not just on feedback quality but also on actual patient-trainee communication skill development must also be addressed in future studies.

### Limitations

While the comments and ratings by both faculty and trainees appear to give a clearly supportive picture, limitations in regards to the precise value of the numerical feedback ratings need to be acknowledged as this was not a formal randomized study. As volunteers, monitoring consultants may have been more invested in education and thus give better feedback. It was a single-centre study performed in a single clinical department. Another limitation is that we only examined written feedback. Verbal in-the-moment feedback may have been provided to the trainees, and arguably would represent a more effective formative feedback process than the written feedback.

## Conclusions

Introducing live audio monitoring of medical student-patient encounters was associated with a generally high quality of written feedback provided by oncology consultants. Using the portable monitoring system was found to be feasible in busy clinics and consultants reported that it gave them more confidence about the feedback they were giving and that it uncovered previously unknown areas of performance deficiencies. The majority of students found that the monitoring-informed feedback was more helpful for their learning as it appeared to facilitate more specific, personal, actionable, and credible feedback. We recommend the use of live audio monitoring as a convenient way of supporting higher quality clinical feedback in clinical settings where video or in-room observation is not possible.

## Data Availability

The datasets used and/or analysed during the current study are available from the corresponding author on reasonable request.

## Abbreviation

CBME: Competency based medical education
